# Clinical Findings and Radiological Evaluation of WHO-Defined Severe Pneumonia Among Hospitalized Children

**DOI:** 10.7759/cureus.33804

**Published:** 2023-01-15

**Authors:** Rahida Karim, Jehanzeb Khan Afridi, Gul-e- Lala, Shah Rukh Yar, Muhammad Batoor Zaman, Behram Khan Afridi

**Affiliations:** 1 Pediatrics Department, Hayatabad Medical Complex Peshawar, Peshawar, PAK; 2 Pediatrics Department, Khyber Teaching Hospital Peshawar, Peshawar, PAK; 3 Medicine, Khyber Medical College, Peshawar, PAK

**Keywords:** noisy breathing, fever, tachypnea, pneumonia, w.h.o

## Abstract

Background: The leading infectious cause of death in children worldwide is pneumonia. Pneumonia claimed the lives of 740,180 kids under the age of five in 2019, accounting for 14% of all fatalities and 22% of deaths in kids between the ages of 1 and 5. Children and families worldwide are affected by pneumonia, but South Asia and Africa have the highest fatality rates.

Objective: This study aims to determine the clinical risk factors and radiological assessment of the World Health Organization (WHO)-defined severe pneumonia in Pakistani hospitalized children.

Material and methods: This cross-sectional study was carried out in the pediatric department of the Hayatabad Medical Complex between January 2021 and December 2021. The study included kids who had a fever, cough, and fast or difficulty breathing between the ages of 2 and 60 months. All of the included clinical pneumonia cases were acquired in the community.

Results: A total of 360 clinically confirmed patients with pneumonia who presented with fever, cough, and fast or difficulty breathing were enrolled. Age ranged between 2 and 60 months, with a mean age of ±31 months. There were 168 (46.7%) males and 192 (53.3%) females. About 232 (64.4%) had radiological pneumonia, while the rest of the pneumonia cases 128 (35.5%) were without a radiological diagnosis. The most common presenting complaint was noisy breathing 119 (33%), followed by refusal of feeds 81 (22.5%), lethargy 69 (19.2%), seizure 40 (11.1%), nasal drainage 29 (8%), and abdominal pain 22 (6.1%).

Conclusion: The most specific clinical finding of radiographic pneumonia was bronchial breathing, while tachypnea was the most sensitive sign.

## Introduction

A severe respiratory infection that damages the lungs is known as pneumonia. The alveoli are stuffed with pus and fluid when someone has pneumonia, which makes breathing difficult and reduces oxygen intake. The leading infectious cause of death in children worldwide is pneumonia [1,2]. Pneumonia claimed the lives of 740,180 kids under the age of five in 2019, accounting for 14% of all fatalities and 22% of deaths in kids between the ages of 1 and 5 [3]. Children and families worldwide are affected by pneumonia, but South Asia and Africa have the highest fatality rates [4].

Chest X-rays continue to be the diagnostic tool of choice in developing countries like Pakistan, and treating physicians frequently employ WHO recommendations to diagnose and treat pneumonia [5]. Since getting the right samples from the lower respiratory tract for culture and microbiological analysis may be challenging, radiography has been regarded as the most accurate tool for diagnosing pneumonia [1,6]. When and how to order a chest X-ray in a case of suspected pneumonia is still up for debate [7].

When bacterial agents, primarily *Streptococcus pneumoniae* and *Hemophilus influenzae*, were the predominant cause of the disease in the patients, prior research revealed the clinical predictors of radiographic pneumonia [8]. The previous WHO recommendations for pediatric pneumonia diagnosis and therapy concentrated on bacterial agents [9].

It is indeed possible that pneumonia's radiographic and clinical symptoms have changed since they were first discovered [10]. This hospital-based study was therefore carried out to evaluate the clinical outcomes and radiological assessment of WHO-defined children hospitalized with severe pneumonia.

## Materials and methods

This cross-sectional study was carried out in the pediatric department of Hayatabad Medical Complex between January 2021 and December 2021. After approval from the Hospital Research and Ethical Committee (IREB) at the Hayatabad Medical Complex in Peshawar under reference number 781/HEC/B&PSC/2022, children between the ages of 2 and 60 months who had a fever, cough, or fast or difficult breathing were included in the study. Clinical pneumonia patients that were included were all caught up in the community. There were no pneumonia cases that occurred in the hospital. Exclusion criteria for study participants included children with a history of foreign body aspiration, chronic respiratory disease (cystic fibrosis/bronchopulmonary dysplasia), known asthma, newly diagnosed asthma (requiring more than one bronchodilator or systemic steroids), and known heart disease. In the present research, the etiological diagnosis (bacterial vs. viral) was not made.

Each child's parents supplied information about their child's immunization history, including information about the Pneumococcal conjugate vaccine (PCV) and *Haemophilus influenzae* type b (Hib) shots. The youngest participant in this trial was two months old and had received PCV-10 and Hib vaccinations at least once. The children were checked by a respiratory doctor for symptoms of tachypnea, nasal flare-up, grunting, decreased air entry, chest indrawing, bronchial breath sounds, and hypoxemia. Hypoxia is defined as low oxygen levels in our body tissues. It causes symptoms like confusion, restlessness, difficulty breathing, rapid heart rate, and bluish skin.

Children aged 2 to 11 months (with 50 or more breaths per minute), as well as 12 to 59 months (with 40 or more breaths per minute), were used as the WHO cut-off criteria to define age-adjusted tachypnea. Based on the X-ray results, radiological and non-radiological pneumonia made up the two categories of clinical pneumonia. Then, it was determined what each clinical predictor's sensitivity and specificity were.

Radiological pneumonia and non-radiological pneumonia were compared using the t-test for continuous variables and the Chi-square test for categorical data. Using SPSS 23.0 (SPSS, Inc., Chicago, IL), statistical analysis was carried out. All of the data were presented in tables and figures. The criterion for statistical significance was a P-value of <0.05.

## Results

Three hundred and sixty clinically diagnosed pneumonia patients who had a fever, cough, or fast or difficult breathing were included. With a mean age of 31 months, the age ranged from 2 to 60 months. There were 192 (53.3%) females and 168 (46.7%) males. The age group was examined, with 115 (31.94%) children between the ages of 2 and 12 months, 81 (22.5%) children between the ages of 13 and 24 months, 73 (20.3%) children between the ages of 25 and 48 months, and 91 (25.3%) children between the ages of 49 and 60 months (Table 1).

**Table 1:** Gender and age group distribution Gender distribution along with age group

Gender/age group	Frequency	Percentage
Gender		
Male	168	46.7%
Female	192	53.3%
Age group		
2–12 months	115	31.9%
13–24 months	81	22.5%
25–48 months	73	20.3%
49–60 months	91	25.3%

**Figure 1:**
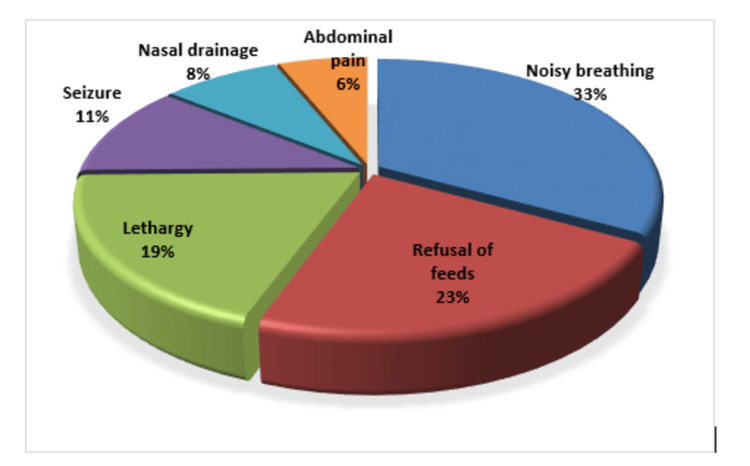
Common presenting complaints Presenting complaints

The most common presenting symptom was noisy breathing, which happened in 119 (33%), followed by a refusal to eat (81.22%), lethargy (69.19%), seizures (40.11.1%), nasal drainage (29.8%), and abdominal pain (22.6%) (Figure 1). 

**Figure 2:**
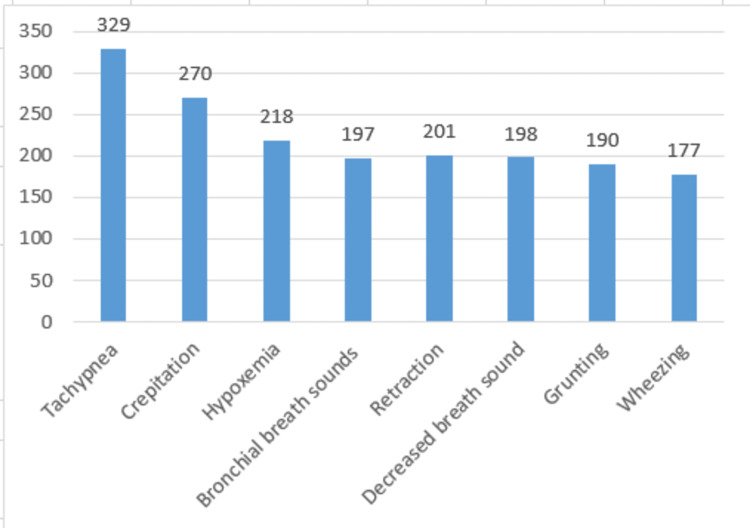
Commonly observed signs

Tachypnea was observed in 329 (91.4%) of the children examined, along with crepitation in 270 (75%), hypoxemia in 218 (60.6%), bronchial breath sounds in 197 (54.7%), grunting in 190 (52.8%), wheezing in 177 (49.2%), decreased breath sounds in 198 (55%), and retraction in 201 (55.8%) (Figure 2).

**Figure 3:**
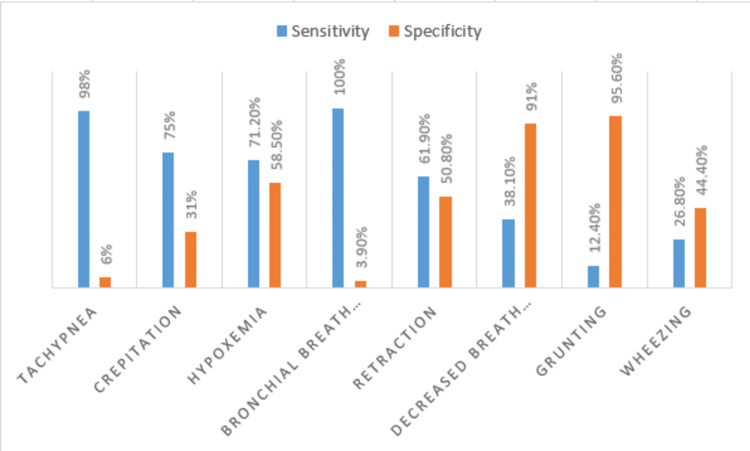
Sensitivity and specificity among clinical features

Tachypnea had a sensitivity and specificity of 98% and 6%, crepitation 75% and 31%, hypoxemia 71.2% and 58.5%, bronchial breathing 100% and 3.9%, retraction 61.9% and 50.8%, decreased breath sound 38.1% and 91%, grunting 12.4% and 95.6%, and wheezing 26.8% and 44.4% sensitivity and specificity, respectively (Figure 3).

**Figure 4:**
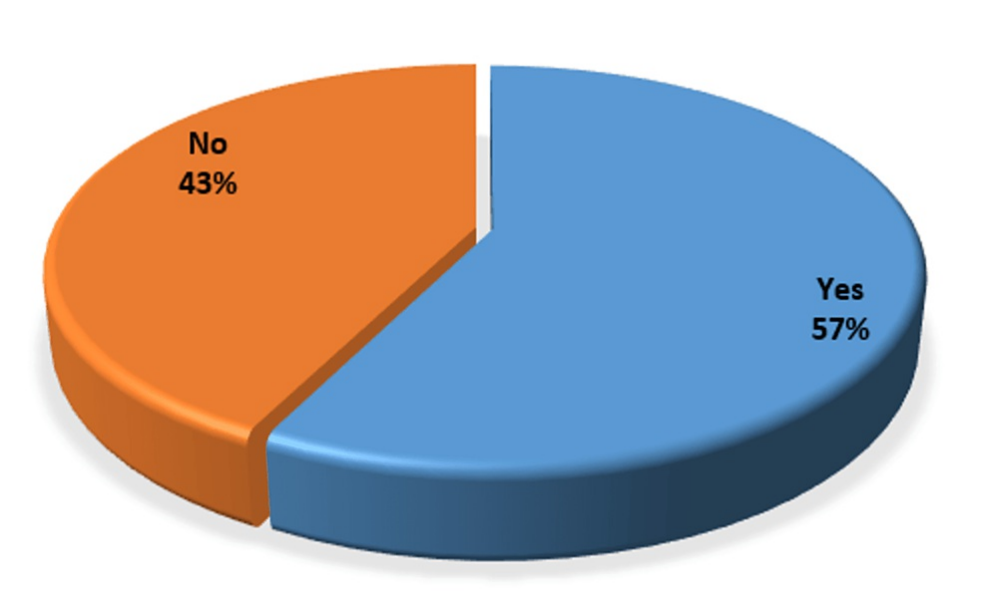
Radiological evaluation of severe pneumonia

There was no radiologic proof of pneumonia in more than half of the children 207 (57.5%) who were clinically diagnosed with severe pneumonia (Figure 4).

Noisy breathing and nasal discharge were clinical symptoms that were significantly associated with radiological pneumonia. Among children, hypoxemia, grunting, wheezing, and decreased breath sounds were the clinical signs that were strongly associated with radiological pneumonia.

## Discussion

Numerous infectious organisms, such as bacteria, fungi, and viruses, can cause pneumonia. Streptococcus pneumoniae is the most common cause of bacterial pneumonia in children, followed by Haemophilus influenzae type b (Hib) [11]. Pneumonia can be spread in several ways. When breathed in, the common viruses and bacteria in a child's nose or throat can infect the lungs. Additionally, airborne droplets from a cough or sneeze might transmit them. Additionally, particularly during and right after childbirth, pneumonia can spread through blood [12]. The various bacteria that cause pneumonia and the means of transmission require further study since they are crucial for both treatment and prevention.

Tachypnea had good sensitivity (98%) but poor specificity (6%) in our study for the identification of radiographic pneumonia. There is a diagnostic issue because most rural health institutions lack X-ray machines, despite the fact that radiography is the gold standard for detecting pneumonia in developing countries like Pakistan. Due to the radiation risks and high frequency of coughs in children, it is also not practical to have every child with a cough have a chest X-ray examination. Therefore, we continue to rely on the simple clinical markers advised by WHO for diagnosing and treating pneumonia [13]. The WHO considers tachypnea to be a sensitive but unreliable sign of pneumonia. Because of this, using tachypnea as the only sign of pneumonia leads to overdiagnosis and antibiotic overprescription. According to Banstola and Banstola [14], radiological pneumonia was present in 64.4% of children, which was quite similar to our findings (57.5%). However, our findings of radiological pneumonia (57.5%) were contrary to the results of Basnet et al., where radiological pneumonia was less common (36%) [15].

Although tachypnea was determined to be the most sensitive indicator of pneumonia in the current investigation (98%), its specificity was poor (6%), and radiological pneumonia was not significantly predictable. Similarly to this, tachypnea failed to discriminate between children who had radiographic pneumonia and those who did not, in research by De Wals et al., despite having a sensitivity of above 95% [16]. Similar to what Scott et al. found, tachypnea had a sensitivity of 74%, and they came to the conclusion that it might be used as a clinical screening indicator for detecting pneumonia in children [17].

In the current study, bronchial breath sounds had a 100% sensitivity for identifying pneumonia among all other indicators. The specificities of grunting and reduced breath sounds were quite excellent, at 95.6% and 91%, respectively, comparable to the study by Lucero et al., who found that decreased breath sounds had a 97% specificity [18].

There is currently no one clinical symptom that can accurately predict radiographic pneumonia. According to the updated WHO definition of pneumonia, children with pneumonia should be diagnosed often in settings with limited resources by looking for tachypnea and/or retractions. According to a research by Ben Shimol et al only 111 of the 324 children diagnosed with radiological pneumonia fit the WHO case definition of pneumonia, proving that the WHO criteria were neither sensitive nor specific in predicting pneumonia in young children [19].

Following adjustment for all the clinical signs and symptoms, only hypoxemia was found to be independently associated with radiological pneumonia, despite the fact that noisy breathing, tachypnea, nasal discharge, wheezing, and decreased breath sounds were all significantly associated with radiological pneumonia on univariate analysis [20].

In this research, hypoxemia as a clinical indicator showed greater sensitivity (71.2%) and specificity (58.5%) for predicting radiological pneumonia. Similar to what Bilkis et al. observed, hypoxemia was a clinical variable in the current study that was strongly linked with radiological pneumonia [21].

Limitations

First, because the current study included children with pneumonia up to the age of 5, this result would not apply to kids older than five; nonetheless, leaving out kids older than five would ignore how pneumonia is becoming more common in this age group, according to epidemiology and clinical symptoms. Second, because this study was carried out at a tertiary care hospital (data were interpreted by a respiratory physician and a radiologist), in an environment where these resources are absent, generalizing the findings may be challenging.

## Conclusions

Hypoxemia was the only independent predictor for radiographic pneumonia, while bronchial breathing was the most distinct clinical finding. The introduction of new vaccines may be the source of this shifting pattern in the clinical presentation and epidemiology of pediatric pneumonia, necessitating a reevaluation of the clinical predictors of pediatric pneumonia. Furthermore, before diagnosing and treating pediatric pneumonia, the physician should take into consideration a variety of clinical variables rather than relying just on one sign or symptom.
